# Plastron appendiculaire: intervention en urgence ou différée: à propos d’une série de 27 cas dans la clinique chirurgicale de l’Hôpital Aristide Le Dantec

**DOI:** 10.11604/pamj.2018.29.15.10038

**Published:** 2018-01-08

**Authors:** Touré Fodé Baba, Wade Thomas Marcel Mbar, Diao Mohamed Lamine, Sylla Mohamed Aly, Tendeng Jacques Noel, Cissé Mamadou, Touré Cheikh Tidiane, Konaté Ibrahima

**Affiliations:** 1 Hôpital Aristide Le Dantec, Dakar, Sénégal; 2 UFR Santé Université de Thiès, Sénégal; 3 Département de Chirurgie et Spécialité Université Gaston de Saint-Louis, Sénégal

**Keywords:** Plastron, appendice, différée, urgence, traitement, Plastron, appendix, deferred, emergency, treatment

## Abstract

Le but de notre étude était d’évaluer les résultats du traitement chirurgicale du plastron appendiculaire après appendicectomie différée ou immédiate. Il s’agit d’une étude rétrospective de type descriptif allant de Janvier 2000 au 31 Décembre 2007 portant sur 27 patients reçus et traités pour plastron appendiculaire. Le diagnostic était clinique par la présence d’une masse à la fosse iliaque droite, échographique, ou per opératoire. Tous les patients opérés en urgence ont été classés dans le groupe I et ceux opérés à froid, groupe II. Nous avons noté 18 hommes et 9 femmes avec un sex-ratio homme /femme = 3. L’âge moyen de nos patients était de 33 ans avec des extrêmes de 19 et 57 ans. Les signes cliniques étaient dominés par la douleur à la fosse iliaque droite et la fièvre respectivement 25 (92,6%) et 15 (55,6%) des cas. Dans le groupe I, l’appendicectomie n’a pu être réalisée pour 7 patients (n = 15) dû aux difficultés opératoires. Dans les autres cas, l’appendicectomie était possible au prix de l’élargissement de l’incision de Mac Burney et une durée de séjour plus longue. Le groupe II a concerné 12 patients (n = 12) avec 9 abords laparoscopiques et dans 3 cas, la voie de Mac Burney a été empruntée. Trois cas d’adhérences péritonéales ont été notés lors de la coelioscopie. L’appendicectomie à froid du plastron appendiculaire est un procédé sûr et efficace. Elle permet d’éviter les cicatrices inesthétiques et les fistules digestives iatrogènes. L’appendicectomie immédiate doit être abandonnée chez les patients porteurs de plastron appendiculaire car elle engendre beaucoup de morbidité.

## Abstract (en)

Our study aimed to evaluate the outcome of the surgical treatment of appendicular plastron after deferred or emergency appendectomy. We conducted a retrospective, descriptive study of 27 patients treated for appendicular plastron from January 2000 to 31 December 2007. Diagnosis was based on clinical examination showing a mass in the right iliac fossa, on ultrasound or made intraoperatively. All patients undergoing emergency surgery were classified in Group I while those undergoing deferred surgery were classified in Group II. 18 men and 9 women were registered, with a sex-ratio man /Woman=2. The average age of patients was 33 years, ranging between 19 and 57 years. Clinical signs were dominated by pain in the right iliac fossa and fever in 25(92.6%) and 15 (55.6%) of cases respectively. In group I, appendectomy couldn’t be performed in 7 cases (n= 15) due to surgical complications. In all other cases appendectomy was performed by enlarging Mac Burney’s incision and was associated with longer length of stay in hospital. Group II included 12 patients (n=12), 9 underwent laparoscopy and 3 patients underwent Mac Burney’s incision. 3 cases with peritoneal adhesions were detected during coelioscopy. Deferred appendectomy of appendicular plastron is a safe and efficient surgical procedure. It allows to avoid unattractive scarrings and iatrogenic digestive fistulas. Emergency appendectomy shouldn’t be performed in patients with appendicular plastron because it increases the risks of morbidity.

## Introduction

Le plastron est une forme évolutive de l’appendicite aiguë dont la fréquence se situe entre 2 et 6% [1]. Il nécessite une prise en charge chirurgicale, il n y a pas encore de consensus sur le protocole thérapeutique. L’appendicectomie peut être immédiate ou différée de quelques semaines [1-5]. Le but de cette étude était d’étudier les aspects épidémiologiques, cliniques et thérapeutiques du plastron appendiculaire.

## Méthodes

Il s’agit d’une étude rétrospective de type descriptif allant de Janvier 2000 au 31 Décembre 2007 portant sur 27 patients reçus et traités pour plastron appendiculaire. Le diagnostic du plastron était établi chez tous les patients, soit cliniquement par la présence d’une masse fébrile et douloureuse à la fosse iliaque droite, échographique et ou la présence à l’intervention d’un plastron appendiculaire évident. Les variables étudiés étaient âge, sexe, les aspects cliniques, parcliniques et les différentes méthodes thérapeutiques (appendicectomie en urgence ou appendicectomie à froid) et les résultats (durée de séjour, morbidité) ont été analysés. Les malades ont été répartis en deux groupes. Tous les patients opérés en urgence ont été classés dans le groupe I et ceux opérés à froid, groupe II. Dans le groupe II: les patients ont bénéficié un traitement médical conservateur avec surveillance rapprochée, antibiothérapie et vessie de glace. Dès que le patient est amélioré, quitte l’hôpital et une appendicectomie secondaire par laparoscopie ou incision de Mac Burney était programmée six à huit semaines plus tard. Le traitement initial conservateur était comportait perfusion paracétamol à la dose 4g/24 H; des antibiotiques par voie intraveineuse (ampicilline 4; la gentamycine 5mg/Kg par jour et le métronidazole 500mg/8h pour 7 à 10 jours). Le relai est assuré par la voie orale jusqu’à la résorption de la masse. Les patients porteurs d’abcès appendiculaire avant le diagnostic du plastron, les tumeurs coecales et la tuberculose digestive ont été exclus de l’étude. Tous nos patients ont eu au moins 3 consultations avec un recul de 4 ans.

## Résultats

En huit ans, nous avons colligé 27 dossiers de plastrons appendiculaires dont 18 hommes et 9 femmes avec un sex-ratio homme/femme = 3. L’âge moyen de nos patients était de 33 ans avec des extrêmes de 19 et 57 ans. Les signes cliniques sont résumés dans le Tableau 1.

**Tableau 1:** Signes cliniques

Signes	Nombres de cas	Fréquence (%)
Douleur à la fosse iliaque droite	25	92, 6
Fièvre > 38° C	15	55,6
Vomissements et / ou nausées	11	40,7
Masse à la fosse iliaque droite	6	22,2
Aménorrhée	2	7,4
Syndrome occlusif	2	7,4
Diarrhée	1	3,7
Constipation	1	3,7

**Paraclinique:** Nous avons noté une hyperleucocytose supérieure à 10000/mm^3^ chez 15 patients (n = 27). L’échographie abdominale a été réalisée chez 18 patients soit 72% des cas et le diagnostic du plastron appendiculaire a été posé dans 12 cas. L’ASP réalisé chez deux patients a objectivé les niveaux hydroaériques de type grêlique.

**Traitement:** Le groupe I a concerné 15 patients (n = 15) et les gestes suivants ont été réalisés: un cas appendicectomie laparoscopique; 7 appendicectomies par voie de Mc Burney. Dans 7 cas, l’appendicectomie n’a été réalisée due au blindage de la masse et aucun cas de crise appendiculaire n’a été noté après un recul de 4 ans. La laparotomie par voie médiane après échec de l’incision de Mac Burney a été réalisée dans un cas pour un appendice pseudo tumoral, rétrocaecal dont la libération a occasionné une brèche iatrogène et réparée secondairement. Nous avons élargi l’incision dans 8 cas. Le groupe II a concerné 12 patients (n = 12) avec 9 abords laparoscopiques et dans 3 cas, la voie de Mac Burney a été empreintée. Trois cas d’adhérences péritonéales ont été notés lors de la coelioscopie.

**Suites opératoires:** Les suites opératoires immédiates ont été simples dans 92,6% (25/27) des cas. Une suppuration rétropéritonéale a été notée chez un patient du groupe I. Le suivi à 3 mois a objectivé un cas de retard de cicatrisation de l’orifice de trocart ombilical pour le groupe II. Aucun cas de décès n’a été enregistré. Trois plaintes pour cicatrices inesthétiques ont été enregistrées après une année de suivi pour les patients groupe I et une douleur résiduelle a été notée chez un patient du groupe II opéré par laparoscopie après un suivi de 2 ans (Tableau 2). La durée moyenne de séjour hospitalier a été de 5 jours. Elle a été longue pour les patients du groupe I (7 jours avec les extrêmes de 4 et 60jours) courte pour ceux du groupe II (3 jours avec les extrêmes de 2 et 6 jours sans différence significative entre la voie de Mac Burney et l’abord coelioscopique). La durée de 60 jours a été observée chez un seul patient qui a eu une suppuration rétro péritonéale.

**Tableau 2:** Suites opératoires

Suites opératoires	Groupe I	Groupe II
Suppuration rétropéritonéale	+	-
Retard de cicatrisation	-	+
Cicatrice inesthétique	+	-
Douleur résiduelle	-	+

Légende: présent (+); absent (-)

## Discussion

Le plastron appendiculaire a une symptomatologie polymorphe. Dans notre étude, les signes cliniques étaient dominés par la douleur à la fosse iliaque droite et la fièvre respectivement 92,6 et 55,6% des cas. Willemsen JP et al. [4] ont rapporté 47% de masse abdominale palpable dans une série de 233 patients et la température moyenne était de 38,2°C. L’intervention immédiate sur le plastron appendiculaire entraîne des difficultés per opératoires avec une morbidité élevée et une durée de séjour hospitalier longue. Dans notre série, l’appendicectomie n’a pas été réalisée pour 7 patients (n = 15) dû aux difficultés opératoires. Dans les autres cas, l’appendicectomie était possible au prix de l’élargissement de l’incision de Mac Burney, de brèche iatrogène ou de laparotomie médiane avec pour corolaire de suppuration retro péritonéale et de cicatrices inesthétiques à ceux-ci s’ajoute la durée de séjour longue et son coût élevé. Keli E et al. ont réalisé 150 appendicectomies laparoscopiques et ont noté 3 cas de conversions sur 4 plastrons opérés [6]. Nous n’avons observé aucune morbidité chez les patients opérés à froid par l’abord de Mc Burney. La majorité des patients opérés à froid l’ont été par laparoscopie. Il n’y avait pas de différence concernant le séjour hospitalier entre la voie laparoscopique et de Mc Burney.

L’abord coelioscopique évite la recherche fastidieuse de l’appendice. Enfin, on estime que la prévalence des brides post opératoires serait nulle après coelio-chirurgie [7,8]. Faut-il proposer aux patients une appendicectomie secondaire après la guérison d’un plastron appendiculaire ? Deux tendances s’opposent: l’abstention et l’appendicectomie systématique. Le principal argument des défenseurs de l’abstention réside dans la mortalité supposée élevée de l’appendicectomie secondaire, alors que les défenseurs de l’appendicectomie « à froid » systématique mettent en avant les risques de récidive et de diagnostics erronés [1, 9]. L’abstention thérapeutique a un risque de récidive 15% [9, 10]. Par ailleurs, Dixon MR. a montré que la récidive était moins sévère que la première poussée [11]. L’analyse histologique des pièces opératoires montrait un appendice non inflammatoire dans 30-38% des cas [11-13]. Cependant une étude a montré que l’appendicectomie secondaire systématique impliquait un accroissement de 38% du coût global de la prise en charge [14].

## Conclusion

L’appendicectomie à froid du plastron appendiculaire est un procédé sûr et efficace. Elle permet d’éviter les cicatrices inesthétiques et les fistules digestives iatrogènes. L’appendicectomie immédiate engendre beaucoup de morbidité. L’abstention thérapeutique n’est préférable que pour les patients à suivi facile. Le patient doit être informé du risque de récidive et les données sur lesquelles est fondée la décision d’abstention.

### Etat des connaissances actuelles sur le sujet

- Le plastron appendiculaire est une complication évolutive de l’appendicite, une péritonite plastique qui se manifeste cliniquement par une masse douloureuse de la fosse iliaque droite avec sensation d’empâtement;
- Le syndrome infectieux est marqué par une fièvre à 38.5°C et une hyperleucocytose marquée;
- L’échographie abdominale et le scanner permettent d’affirmer le diagnostic par la présence d’une masse de la fosse iliaque droite engainant les anses grêles avec les signes inflammatoires; il n’y a pas de consensus pour sa prise en charge; en l’absence de traitement ou le traitement inefficace, l’évolution se fait en général vers l’abcédation.

### Contribution de notre étude à la connaissance

- Cette étude nous a permis de comparer 2 méthodes thérapeutiques du plastron appendiculaire et de formuler des recommandations;
- L’appendicectomie à froid du plastron appendiculaire est un procédé sûr et efficace; elle permet d’éviter les cicatrices inesthétiques et les fistules digestives iatrogènes;
- L’appendicectomie immédiate engendre beaucoup de morbidité alors, elle doit être abandonnée.

## Conflits d’intérêts

Les auteurs ne déclarent aucun conflit d’intérêts.

## Contributions des auteurs

Tous les auteurs ont lu et approuvé la version finale du manuscrit.
